# A Rare Case of Splenic Artery Aneurysm With Unusual Combination of Pancytopenia and Massive Splenomegaly in a Tertiary Care Hospital

**DOI:** 10.7759/cureus.47940

**Published:** 2023-10-30

**Authors:** Shrawani Kulkarni, Swarupa Chakole, Tanishq Dubey, Seema Yelne

**Affiliations:** 1 Community Medicine, Jawaharlal Nehru Medical College, Datta Meghe Institute of Higher Education and Research, Wardha, IND; 2 General Medicine, Jawaharlal Nehru Medical College, Datta Meghe Institute of Higher Education and Research, Wardha, IND; 3 Nursing, Shalinitai Meghe College of Nursing, Datta Meghe Institute of Higher Education and Research, Wardha, IND

**Keywords:** rupture of splenic artery cases, computed tomography, splenomegaly, portal hypertension, splenic artery aneurysm

## Abstract

Aneurysms of the splenic artery leading to extrahepatic portal hypertension are sporadic and infrequently encountered. They typically manifest as a consequence of thrombus or embolus formation. A splenic artery aneurysm (SAA) represents a localized expansion in the diameter of the splenic artery and is one of the most prevalent forms of visceral artery aneurysms. This artery dilation is primarily attributed to pancreatitis, trauma, or atherosclerosis, commonly affecting elderly patients. Patients affected by this condition typically remain asymptomatic, except for an audible bruit over the aneurysm site, unless a rupture occurs. In the event of a rupture, early indicators include abdominal pain, hemoperitoneum, and a positive Kerr sign, all indicative of SAA rupture. Most SAAs are incidentally discovered, with CT angiography being the preferred diagnostic tool. We present the case of a 38-year-old female (gravida 1, para 1) with a previous full-term normal delivery, who presented to a rural tertiary care hospital with a two-week history of left-sided abdominal pain. A CT scan of the abdomen revealed a solitary aneurysm in the distal portion of the splenic artery, accompanied by perisplenic fluid collection resulting in splenomegaly. Given the critical risk of rupture, which can result in life-threatening bleeding, prompt and accurate diagnosis assumes paramount significance. It is worth noting that the diagnosis of SAA often occurs incidentally due to its asymptomatic nature in its early stages. We document this unique occurrence of extrahepatic SAA contributing to pancytopenia, portal hypertension, and extensive splenomegaly to provide valuable insights for medical professionals in recognizing and managing such presentations. This awareness can help prevent unnecessary diagnostic and therapeutic interventions.

## Introduction

Splenic artery aneurysms (SAAs) are infrequent vascular anomalies characterized by localized dilation of the splenic artery, presenting unique clinical challenges. While SAAs have been documented in various clinical contexts, their association with extrahepatic causes and the concurrent development of distinctive hematological complications, such as pancytopenia, remain rare and underexplored [1]. True SAAs are uncommon, with an estimated prevalence ranging from 0.02% to 0.1%. Giant aneurysms are more infrequent, with only approximately 20 documented cases [2]. Aneurysms of the splenic artery manifest in approximately 0.1% of adults. It is estimated that 6% to 10% of such SAAs are predisposed to rupture, with a notable 25% to 40% of these ruptures transpiring during pregnancy, particularly within the third trimester [3]. This case report highlights an exceptional clinical scenario in which an SAA is attributed to an extrahepatic etiology while co-occurring with massive splenomegaly and pancytopenia. This presentation's rarity underscores the importance of early diagnosis and expeditious intervention in managing these intricate and potentially life-threatening cases. By shedding light on this uncommon combination of clinical manifestations, we aim to contribute to the comprehension and effective management of SAAs in unconventional clinical settings.

Intriguingly, SAAs are an uncommon vascular pathology, and their prevalence in the Indian context has been notably limited, potentially due to data collection or awareness gaps. The underlying causes of visceral aneurysms, including SAAs, typically remain elusive, with recent research suggesting that various factors, including the weakening of arterial walls, contribute to the development of true aneurysms. However, the precise pathophysiology of SAAs continues to elude complete understanding, making them a compelling subject of investigation [4].

Furthermore, SAAs present a formidable clinical challenge, particularly due to their propensity for rupture, which can result in significant morbidity and mortality. SAAs are most commonly (60%) associated with a high mortality rate of 25% in case of aneurysm rupture. This increases disproportionately to 75% among pregnant women with a fetal mortality of 95% [5]. Non-pregnant patients who experience SAA rupture often face mortality rates ranging from 25% to 40%, underscoring the gravity of the condition [6]. Tragically, pregnant individuals with SAA carry the highest risk of rupture, frequently leading to devastating outcomes for both mother and fetus. The increased utilization of diagnostic imaging procedures has led to the incidental discovery of SAAs. Still, immediate referral to surgical or interventional radiological expertise remains paramount to mitigate the imminent risk of rupture [7].

The advent of advanced imaging techniques and the presence of skilled diagnostic professionals in rural tertiary care hospitals offer a glimmer of hope for patients facing these life-threatening circumstances. Notably, aneurysms larger than 2 cm in diameter warrant immediate consideration for intervention, given the heightened risk of rupture [8]. The array of available treatment modalities, including open surgery, endovascular approaches, and minimally invasive laparoscopic methods, ensures a tailored approach to each patient's unique clinical scenario. The laparoscopic approach, in particular, offers several advantages, including accelerated recovery, reduced hospitalization duration, and diminished postoperative discomfort. Importantly, even pregnant individuals with SAA may find the laparoscopic method a safe and effective option [9].

Given this backdrop, this case report emerges as a rare and valuable contribution to the medical literature, given its unusual combination of extrahepatic SAA etiology, pancytopenia, and massive splenomegaly [10]. It is a poignant reminder of the critical significance of timely diagnosis and intervention in managing these intricate and life-threatening cases. By sharing our experience with this unique clinical presentation, we aspire to enhance the understanding and management of SAAs, particularly in atypical clinical contexts, ultimately striving to improve patient outcomes and quality of life.

## Case presentation

A 38-year-old woman sought medical attention at the outpatient surgery clinic due to recurrent left upper quadrant pain episodes that had been occurring over the past month. These episodes were characterized by sudden, sharp, stabbing pain lasting less than six hours and severely impacting her breathing ability. Notably, there was no association with nausea or vomiting, but she did report a change in her bowel habits. Additionally, she had experienced weight loss, which coincided with alterations in her bowel habits. Despite taking multiple courses of analgesics and antibiotics, her pain persisted. She had undergone treatment for uterine prolapse 20 days before seeking medical help, and she had no other significant medical history or addiction history.

During her latest severe pain episode, she required hospitalization in the surgery ward for further evaluation. She had previously received initial care and treatment at another hospital. Upon examination, the patient was afebrile, with no systemic abnormalities, and her vital signs were stable, with a pulse rate of 56 beats per minute, blood pressure of 120/70 mmHg, and a respiratory rate of 23 cycles per minute. Laboratory tests indicated a hemoglobin level of 9 g/dL, a total white blood cell count of 4,000/cumm, a platelet count of 2.4 lakhs/cumm, a urea level of 20, and a creatinine level of 0.5 mg/dL. She received a transfusion of one unit of packed red cells. Due to the persistence of abdominal pain, she was referred to our hospital for further evaluation and treatment.

Upon admission to the facility, she experienced another episode of left upper quadrant abdominal pain, leading to her admission to the surgery department. Physical examination revealed a generally healthy musculoskeletal and digestive system. The patient appeared pale, with a body temperature of 37°C, a pulse rate of 47 beats per minute (regular and of good volume), a blood pressure of 110/70 mmHg, and a respiratory rate of 20 breaths per minute. Abdominal examination revealed mild tenderness, guarding, rigidity, and grade III splenomegaly.

Complete blood count results indicated a hemoglobin level of 7.5 g/dL, a white blood cell count of 3.1×10^9^/L, a platelet count of 1.1×10^9^/L, and a red blood cell count of 3×10^12^/L, with an erythrocyte sedimentation rate (ESR) of 10 mm/h. Notably, there was a gradual decline in platelets, leukocytes, and red blood cells. Treatment commenced with intravenous fluids, tranexamic acid, diclofenac, and paracetamol, and a transfusion of one unit of packed red cells. Urine examination and serum amylase levels were within the normal range; all other laboratory tests yielded normal results.

**Figure 1:**
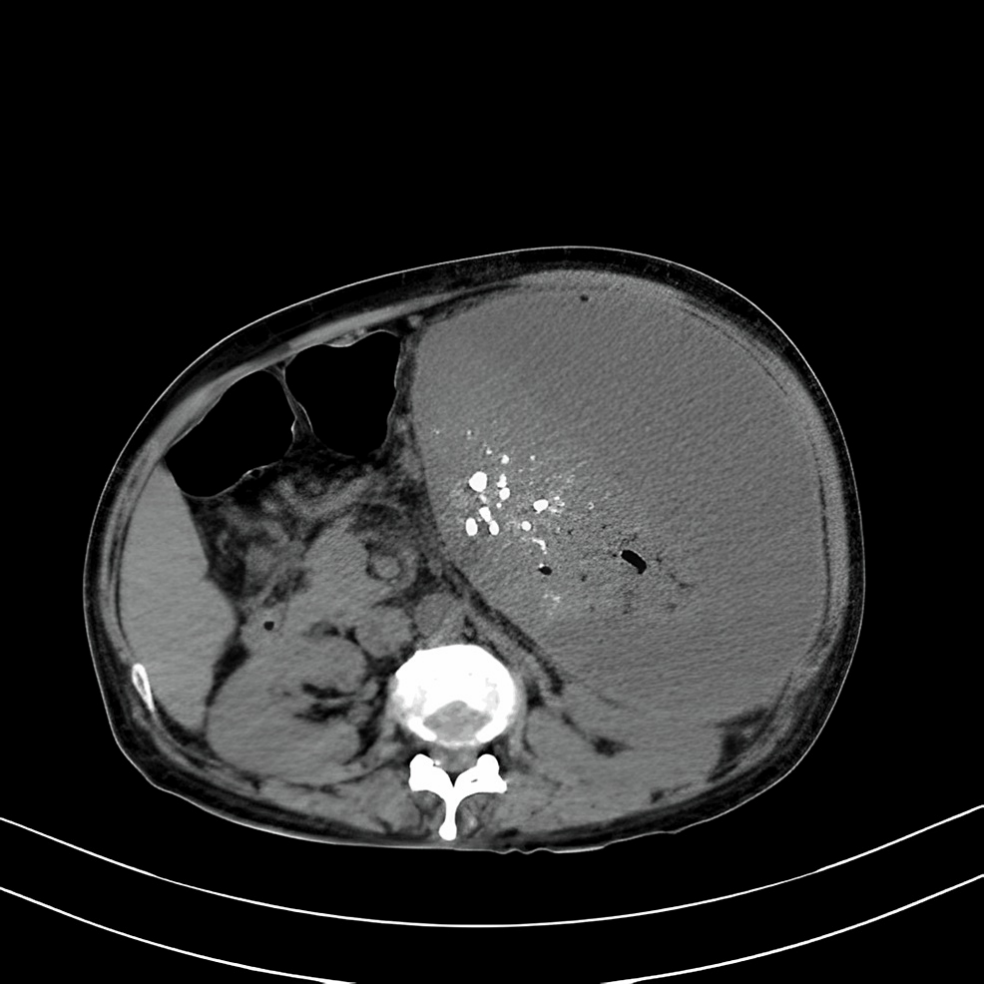
Non-contrast axial image of spleen showing multiple hyperdense focus and air density foci within splenic parenchyma, with large well-defined perisplenic collection.

**Figure 2:**
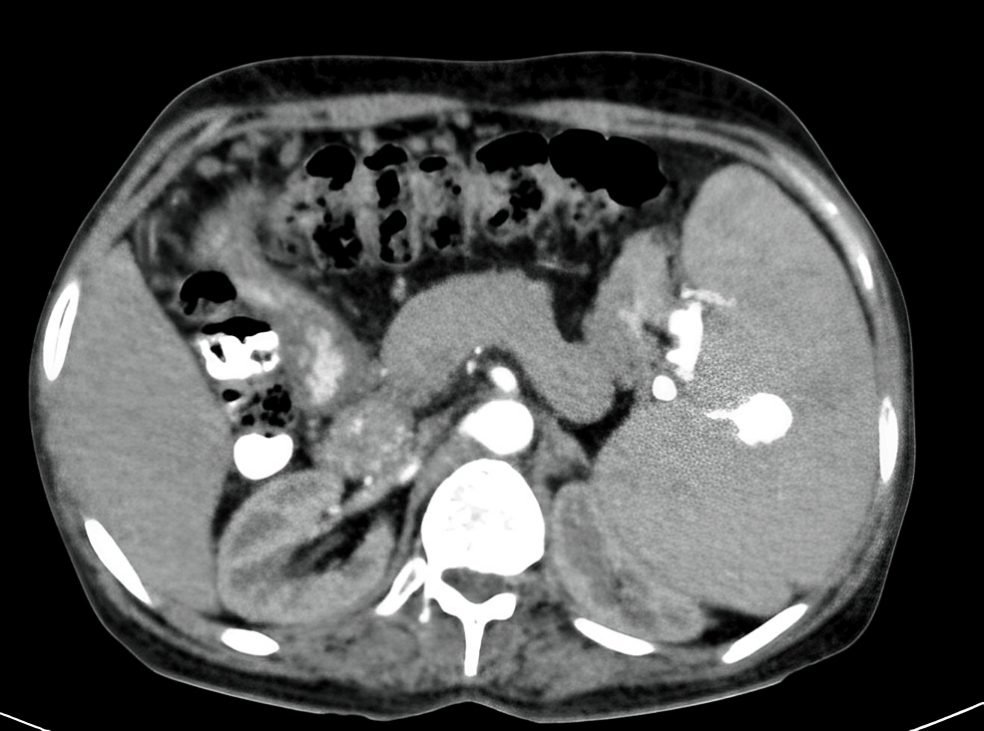
Arterial phase contrast-enhanced axial image of the spleen showing splenomegaly with pooling of blood in spleen and splenic artery aneurysm

Further diagnostic investigation via ultrasound revealed a significant perisplenic collection with internal echoes and multiple echogenic foci within the splenic parenchyma, coupled with splenomegaly measuring 16.7 cm below the left costal margin, which appeared firm and non-tender (Figure 1). No hepatomegaly was observed. CT of the abdomen confirmed the presence of multiple aneurysms in the distal half of the splenic artery, with no thrombus formation and a dilated and tortuous splenic artery (Figure 2).

**Figure 3:**
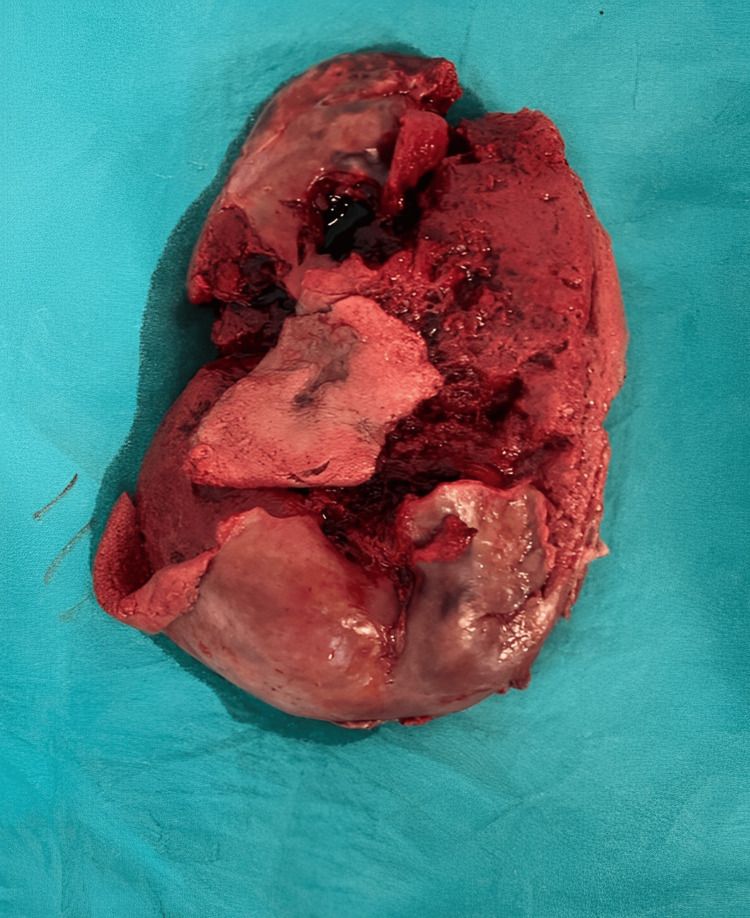
Cross-section of the spleen showing embolus in the splenic artery aneurysm

Given the risk of rupture associated with this condition, emergency surgery was conducted after obtaining informed consent. Laparotomy was performed via an upper abdominal midline incision, adhering to strict aseptic protocols. Under local anesthesia, gastrointestinal visceral arterial embolization (splenic artery embolization) surgery was performed by accessing the right common femoral artery and cannulating the celiac artery. The embolization procedure involved PVA particles (contour: 355-500 microns) and glue, delivered through a Progreat microcatheter. A completed angiogram confirmed the complete occlusion of the aneurysmal sac. Splenectomy revealed a perisplenic blood collection of 500-600 mL, with adhesions between the omentum and the spleen's capsule. During the operative procedure, the descending colon adhered to the splenic capsule (Figure 3). The patient tolerated the procedure well and had no complications during surgery. Subsequently, she was transferred to the Surgical Intensive Care Unit (SICU).

The patient's postoperative course was uneventful, with no surgical complications observed. Ten days after surgery, she was discharged. She was advised to follow up regularly to monitor her condition. During these routine follow-ups, the patient reported no pain, and her blood pressure remained within the normal range for her age and height. Two weeks after discharge, improvements were noted in her blood parameters, including hemoglobin (10.3 g/dL), platelet count (3.5 lakhs/cumm), urea (24 mg/dL), creatinine (0.8), sodium (146 mg/dL), and potassium (4.3 mg/dL). She continued to be pain-free during her regular follow-up visits.

## Discussion

Cirrhotic portal hypertension is a significant risk factor for developing SAAs. A study conducted by Mr. Hajime Sunagozaka emphasized the increased prevalence of SAAs associated with liver cirrhosis, which is more common. Interestingly, this differs from the current case, where the primary cause appears extrahepatic. Additionally, the study noted that giant SAAs are more frequently observed in males, a pattern not observed in the present case [11].

Another case study conducted by Siddharth Yadav focused on giant SAAs and their unique surgical challenges in female patients. This particular challenge was not encountered in the discussed case [2]. Dr. Muwaffaq Mezeil Telfah's study on the pre-rupture diagnosis of SAAs highlighted that all three blood cell counts (RBCs, WBCs, and platelets) remained normal in their cases. In contrast, the discussed case featured pancytopenia, indicating a notable difference in the presentation [12].

WSL De Silva's research depicted SAAs presenting multiple episodes of upper gastrointestinal bleeding, emphasizing the possibility of double or multiple ruptures, especially when patients falsely show hemodynamic stability. However, no such complications occurred [13]. Mr. Chihiro Yoshikawa's study described a case of a giant SAA rupturing into the stomach, leading to hemorrhagic shock and requiring emergency surgery. The importance of immediate diagnosis and treatment was highlighted in preventing catastrophic complications. Fortunately, management occurred before such severe complications arose in the discussed case [14]. Finally, Akshatha Rao Aroor's study on multiple SAAs with portal hypertension and splenomegaly found that extrahepatic presentations can be effectively treated with proper interventions. While the present case involves a single aneurysm, it shares similarities with the studied cases, particularly in portal hypertension and massive hypersplenism [15].

## Conclusions

In conclusion, SAAs remain relatively rare in India, possibly due to limited data collection. The exact cause of visceral aneurysms, including SAAs, remains unknown, but various factors contribute to their development. Rupture of SAAs carries a high mortality rate, particularly during pregnancy. Advancements in imaging and diagnostic capabilities in rural tertiary care hospitals provide hope for early detection and intervention. Aneurysms more significant than 2 cm in diameter should be promptly treated to mitigate the risk of rupture. Treatment options include open surgery, endovascular approaches, and minimally invasive laparoscopic methods. Laparoscopy offers distinct advantages, including quicker recovery, shorter hospital stays, and reduced postoperative pain, even for pregnant patients. This unique case underscores the urgency of early diagnosis and treatment, especially when faced with life-threatening complications such as pancytopenia and massive splenomegaly. Timely intervention can significantly improve a patient's quality of life and outcomes.
